# Concurrent molecular characterization of sand flies and *Leishmania* parasites by amplicon-based next-generation sequencing

**DOI:** 10.1186/s13071-022-05388-3

**Published:** 2022-07-22

**Authors:** Abedelmajeed Nasereddin, Suheir Ereqat, Amer Al-Jawabreh, Mohamad Taradeh, Ibrahim Abbasi, Hanan Al-Jawabreh, Samer Sawalha, Ziad Abdeen

**Affiliations:** 1 Biochemistry and Molecular Biology Department, Faculty of Medicine, Al-Quds University, Abu Deis, East Jerusalem, Palestine; 2 Department of Medical Laboratory Sciences, Faculty of Allied Health Sciences, Arab American University, Jenin, Palestine; 3 Leishmaniases Research Unit, Jericho, Palestine; 4 Al-Quds Nutrition and Health Research Institute, Al-Quds University, East Jerusalem, Palestine; 5 AL-Quds Public Health Society, East Jerusalem, Palestine; 6 Department of Biology and Biotechnology, Al-Quds University, East Jerusalem, Palestine; 7 Ministry of Health, Ramallah, Palestine

**Keywords:** *Leishmania*, Phlebotomine sand flies, Amp-NGS, Taxonomy

## Abstract

**Background:**

Phlebotomine sand flies are vectors of *Leishmania* parasites, which are the causative agents of leishmaniasis. Herein, we developed an amplicon-based next-generation sequencing (Amp-NGS) to characterize sand flies and *Leishmania* parasites simultaneously targeting partial fragments of 18S rDNA and ITS1 genes, respectively.

**Methods:**

Our assay was optimized using reference sand fly (*n* = 8) and *Leishmania* spp. (*n* = 9) samples and validated using wild-caught sand flies from Palestine. The assay was highly specific, and all DNA references were successfully identified to the species level.

**Results:**

Among the wild-caught sand flies (*n* = 187), *Phlebotomus* spp. represented 95% of the collected samples (177/187), including *Ph. sergenti* (147/187, 79%), *Ph. papatasi* (19/187, 10.2%), *Ph. perfiliewi* (3/187, 1.6%), *Ph. tobbi* (2/187, 1.2%) and *Ph. syriacus* (6/187, 3.2%). *Sergentomyia* spp. represented only 5% (10/187) of the collected samples and included *S. dentata* (*n* = 6), S*. fallax* (*n* = 2), *S. schwetzi* (*n* = 1) and *S. ghesquiere* (*n* = 1). The study observed strong positive correlation between sand fly identification results of the Amp-NGS and morphological identification method (*r* = 0.84, df = 185, *P* < 0.001). Some discrepancies between the two methods in the identification of closely related species (i.e. *Ph. perfiliewi*, *Ph. tobbi* and *Ph. syriacus*) were observed. *Leishmania* DNA was detected and identified as *L. tropica* in 14 samples (14/187, 7.5%).

**Conclusions:**

Our assay was sensitive to detect (limit of detection was 0.0016 ng/reaction) and identify *Leishmania* DNA in sand flies, thus representing a new tool for studying sand flies and their associated *Leishmania* parasites in endemic areas.

**Graphical Abstract:**

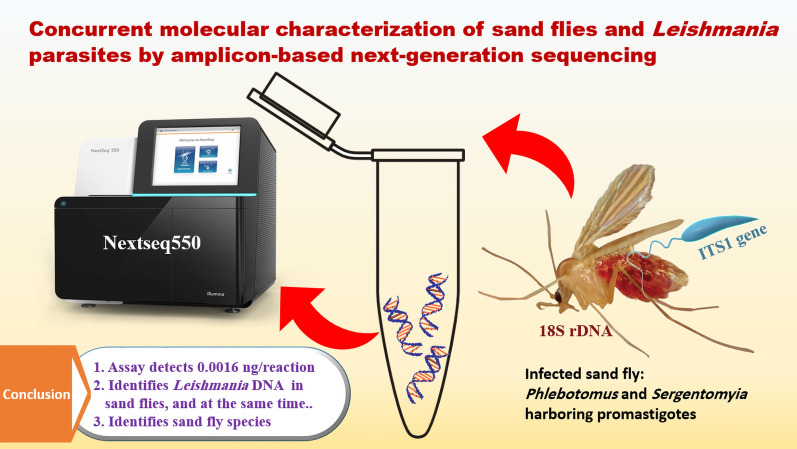

**Supplementary Information:**

The online version contains supplementary material available at 10.1186/s13071-022-05388-3.

## Background

Leishmaniasis is a group of diseases that affect millions of people worldwide, causing different clinical signs, ranging from self-limiting skin lesions to death. Among *Leishmania* spp. described worldwide, about 20 cause disease in humans, including cutaneous leishmaniasis (CL), visceral leishmaniasis (VL) and mucocutaneous leishmaniasis (MCL) [1]. These forms of leishmaniasis may overlap in distribution. For instance, Palestine is a traditional focus of both CL and VL. In particular, cases of human VL and canine leishmaniasis (CanL) caused by *Leishmania infantum* are sporadically reported, whereas CL caused by *Leishmania tropica* and *Leishmania major* is distributed all over the Palestinian districts except in Gaza Strip, which is free from the disease [2–6].

*Leishmania* parasites are transmitted through the bite of infected phlebotomine sand flies (Diptera: Psychodidae). Thus, studying *Leishmania* parasites and their associated sand fly vectors in endemic areas is pivotal in leishmaniasis control, as well as in understanding the transmission dynamics of these parasites in high-risk areas [7–11].

Several phlebotomine sand flies are regarded as vectors for *Leishmania* parasites [12–23], including *Phlebotomus neglectus* (vector of *L. infantum*) [12, 13], *Phlebotomus sergenti* (vector of *L. tropica*) and *Phlebotomus papatasi* (vector of *L. major*) [14]. Besides, the detection of *L. infantum* and *L. major* DNA in *Sergentomyia* spp. may suggest that these species could acquire the parasites while feeding on infected hosts such as rodents [15–18]. However, their vector competences and ability to become infected and to transmit these parasites to other naïve hosts have not been proven yet [15–18].

Palestine is a classical focus of CL and VL. Cases of human VL (hVL) and canine leishmaniasis (canL) caused by *Leishmania infantum* are sporadically reported. Cases of CL caused by *L. tropica* and *L. major* are distributed all over the Palestinian districts except in Gaza Strip, which is free from the disease [7–11]. Several sand fly species are regarded as proven vectors for *Leishmania* parasites such as *Phlebotomus neglectus*, a proven vector of *L. infantum* [12, 13]; *Phlebotomus sergenti*, a vector of *L. tropica*; and *Ph. papatasi*, a vector of *L. major* [14]. Besides, detection of *L. infantum* and *L. major* DNA in *Sergentomyia* species may suggest that these species could acquire the parasites while feeding on infected hosts such as rodents [15]. However, their vector competences and ability to become infected and to transmit these parasites to other naïve hosts have not been proven yet [15–18]. Sand flies of the genus *Sergentomyia* are present throughout the Middle East including Palestine, but without any signs of vector competence [19, 20] such as in Greece and Cyprus [21–23].

Phlebotomine sand fly species identification is traditionally based on microscopic examination of key morphological features [7–11]. This method is time-consuming and requires specific skills for specimen preparation and identification [24]. Molecular techniques targeting several ribosomal, mitochondrial and nuclear DNA have been increasingly used for the identification of sand fly species [24–30]. In a similar manner, several targets have been used for *Leishmania* parasite detection and identification in sand flies, including the kinetoplast DNA (kDNA), internal transcribed spacer 1 (ITS1) region [31–33], and heat shock protein 70 (hsp70) [34]. PCR assays employing these targets present variable degrees of sensitivity and specificity. For instance, most kDNA-based PCR usually present high sensitivity but low specificity. In this regard, ITS1-based PCR-RFLP (PCR-restriction fragment length polymorphism) has been used for detection and identification of Old World *Leishmania* spp. [31–34].

Next-generation sequencing (NGS) technologies have several advantages over classical molecular-based methods, including the ability to generate large quantities of DNA sequences in a single run for in-depth detection of pooled DNA in several DNA samples [35]. High-throughput screening makes DNA sequencing cheaper, faster and more reliable. Recently, Abbasi et al. [36] used this technology for *Leishmania* and sand fly identification, using ITS1 gene and cytochrome oxidase I, respectively. In the present study, we developed a novel amplicon-based NGS (Amp-NGS) method for the identification of *Phlebotomus* and *Sergentomyia* species with concomitant detection and identification of *Leishmania* spp. in these sand flies.

## Methods

### Sand fly collection and DNA extraction

Sand fly collections were made between May and September 2019 using CDC light traps (John W. Hock USA). Two traps per den for three dens were placed in two regions (Al-Khalil and Tubas) where *L. major*, *L. tropica* and *L. infantum* have been reported [8, 37]. All traps were left in the field from dusk to dawn for 3 consecutive nights (36 h). Sand flies were sorted using a Discovery V12 stereomicroscope (Zeiss, Germany). Female sand flies were separated and identified using the taxonomic keys [38–41]. All samples were preserved individually in 1.5-ml micro-tubes containing 70% ethanol. The female collection was subjected to DNA extraction while male samples were kept frozen for future study. Genomic DNA was extracted using a phenol-chloroform method [42]. The DNA was eluted in 50 µl of 1× TE buffer (Tris 10 mM, 1 mM EDTA, pH ~ 8) and quantified by NanoDrop 1000 (Thermo Scientific, USA) and stored at 4 °C until use.

### Reference DNA samples

DNA samples from sand flies of the genera *Phlebotomus* (i.e. *Ph. sergenti, Ph. perfiliewi, Ph. syriacus, Ph. papatasi, Ph. tobbi, Ph. argentipes*), *Sergentomyia* (*S. schwetzi*) and *Lutzomyia* (*L. guyanensis)* were used as DNA standards. The samples were kindly provided by the Prof. Alon Warburg, entomology laboratory (Jerusalem). The samples were identified by microscopic examination and/or by PCR targeting the cytochrome oxidase subunit I (COI) gene The GenBank accession numbers are: *Ph. papatasi* (JX105037), *Ph. sergenti* (JX105039.1), *Ph. argentipes* (JX105038.1). Moreover, DNA samples from different *Leishmania* spp. (three samples of *L. tropica*, two *L. major*, two *L. infantum*, one *L. donovani* and one *L. aethiopica*) were also used in this study (Additional file 1: Table S1).

### Target genes, primers and probes

**Fig. 1:**
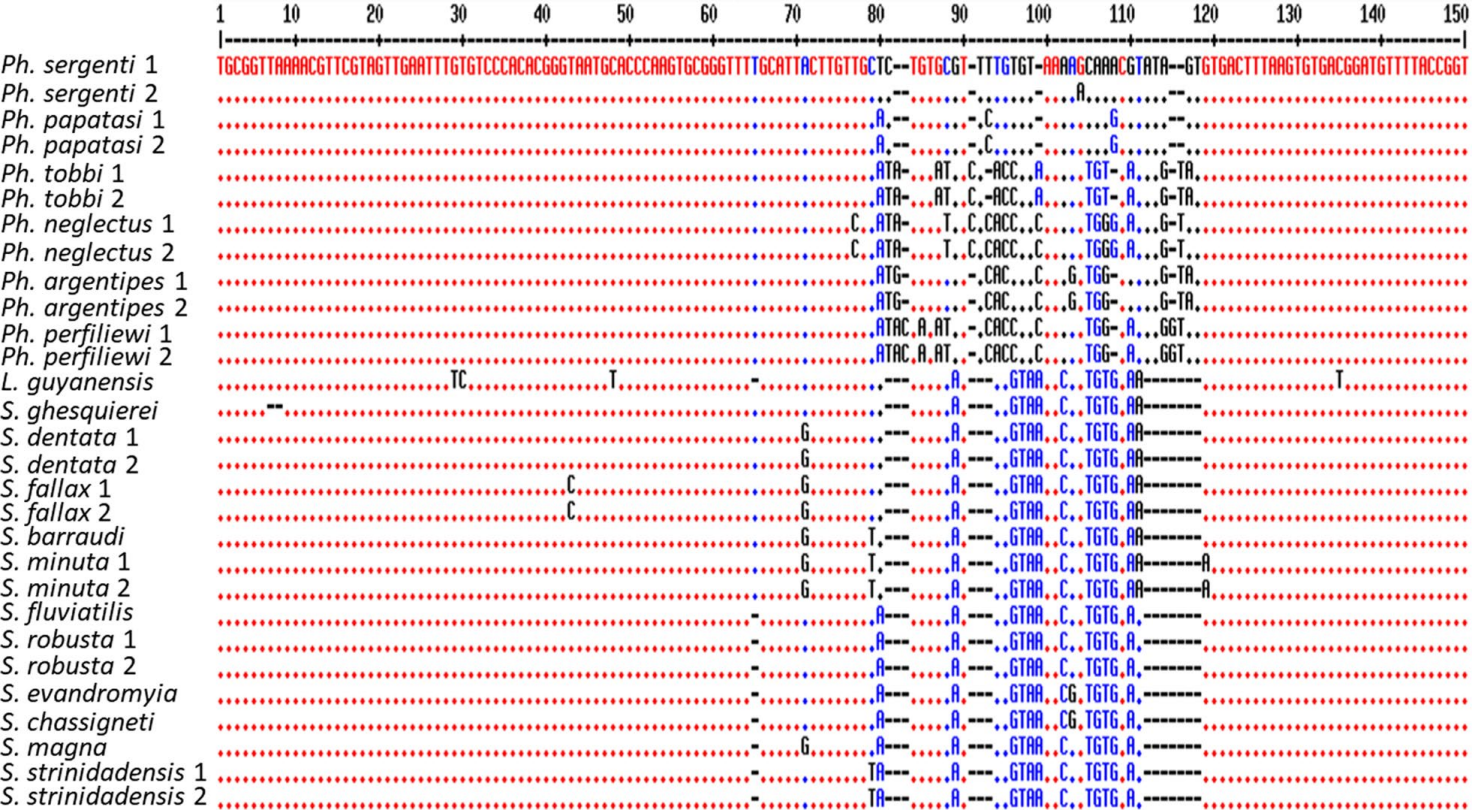
Multiple sequence alignment of the 18S rDNA nucleotide sequences of several sand fly species for designing reverse primer and species-specific probes. Red fonts represent the identical sequences, while blue and black show the differences between the sand fly species that were used for virtual probe selection to identify the sand fly species

**Fig. 2:**
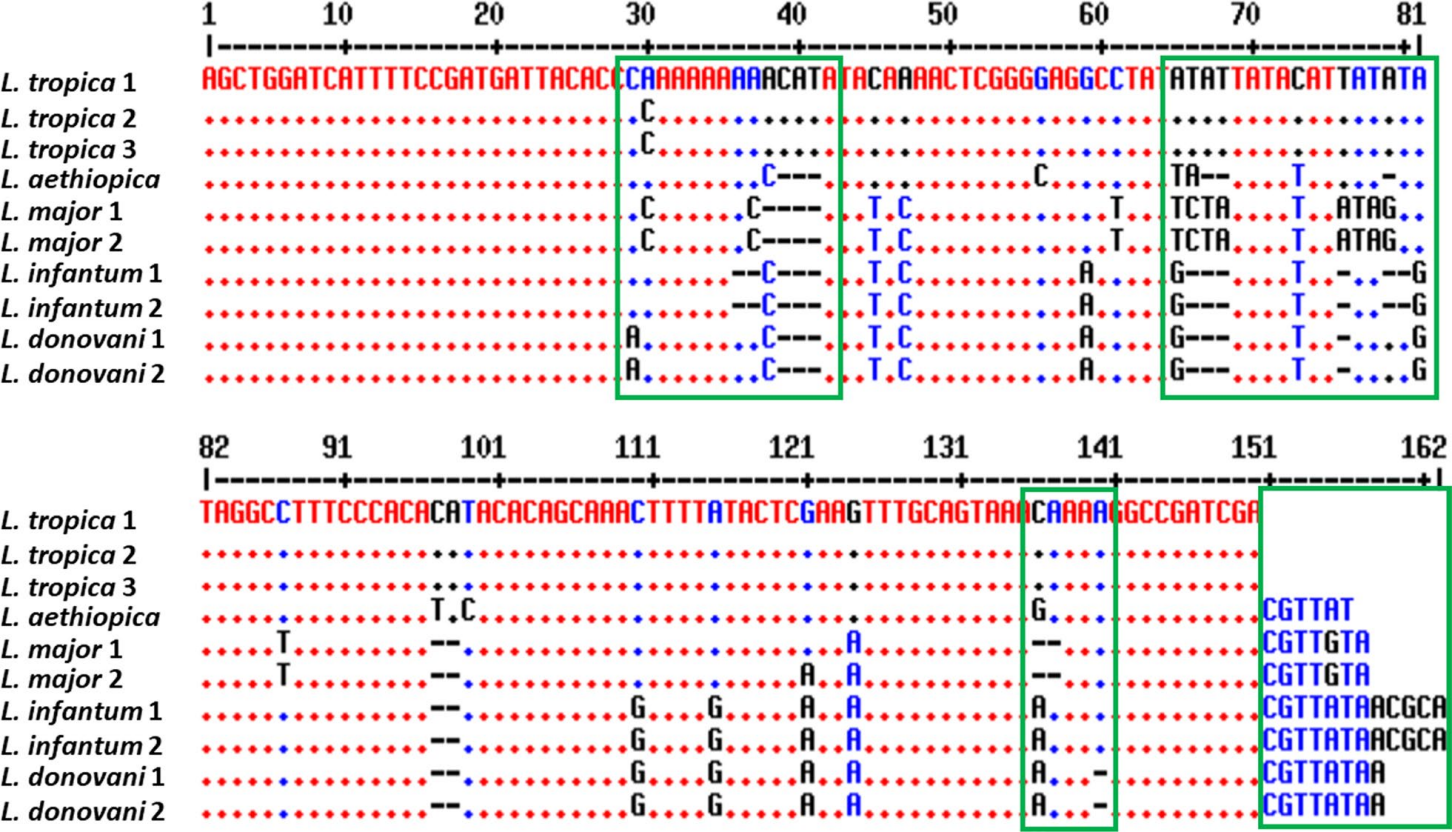
Multiple sequence alignment of the ITS1 nucleotide sequences of several *Leishmania* species to design species-specific probes as indicated in the green boxes. *L. tropica* 1, 2 (Israeli), 3 from Iran, *L. aethiopica* from *Ethiopia*, *L. major* 1 from Ashkhabad (5ASKH), *L. major* 2 from Iraq, *L. infantum* 1 and 2 from Tunisia and France, respectively, *L. donovani* 1 and 2 from India and Sudan, respectively

To design primers for sand fly identification, representative 18S rDNA sequences (*n* = 29) were retrieved from GenBank, used in FASTA format and aligned using the multiple sequence alignment online program (http://multalin.toulouse.inra.fr/multalin/) [43] to identify the conserved and polymorphic regions. At least two DNA sequences from the same species but with different accession numbers were included to check the stability of the polymorphic regions among the species. A conserved region in the 18S rDNA was selected to design primers able to detect different sand fly genera and species. Within the selected 18S rDNA region, a polymorphic 150 bp DNA sequence was chosen to differentiate sand flies to the species level. The forward primer was used as described elsewhere [44] while the reverse primer was selected using primer3 (http://bioinfo.ut.ee/primer3-0.4.0/) (Fig. 1). Sand fly 18S rDNA sequences used in this study and their GenBank accession numbers are shown in Additional file 1: Table S2. Likewise, the ITS1 region (~ 300 bp) was chosen for *Leishmania* spp. detection and identification as described previously [33]. In brief, 13 ITS1 sequences were retrieved from the GenBank (Additional file 1: Table S2), representing different geographic regions to target the conserved species-specific sites (Fig. 2). All primers were modified by adding the Illumina overhang adapter sequences at the 5’ end to fit with Illumina platform NGS system. Primer names, sequences and the expected size of the amplified products are shown in Table 1. Polymorphic sequences in both targets (18S rDNA and ITS1) were used to create virtual specific probes that can be used in our workflow bioinformatics analysis to identify the sand fly and *Leishmania* species using the Galaxy, a free online program (https://usegalaxy.org/). Probes are shown in Additional file 1: Table S3.

**Table 1:** Primer sequences and corresponding target genes used in the study

Primer name	DNA sequences	Target gene /PCR product size	References
ITS1219NGSF	TCGTCGGCAGCGTCAGATGTGTATAAGAGACAGAGCTGGATCATTTTCCGATG	*Leishmania* ITS1/343 bp	[33]
ITS1219NGSR	GTCTCGTGGGCTCGGAGATGTGTATAAGAGACAGATCGCGACACGTTATGTGAG		
SFNGSF	TCGTCGGCAGCGTCAGATGTGTATAAGAGACAGTGCGGTTAAAACGTTCGTAG	Sand flies 18S rDNA/230 bp	[44]
SFNGSR	GTCTCGTGGGCTCGGAGATGTGTATAAGAGACAGACCGGTAAAACATCCGTCAC		This study

### Multiplex PCR for detection of *Leishmania* and sand fly DNA

For simultaneous detection and identification of sand fly and *Leishmania* DNA, two primer sets were used for multiplex PCR, ITS1NGSF/ITS1NGSR and SFNGSF/SFNGSR, which amplified a fragment of 343 bp of *Leishmania* spp. ITS1 and a fragment of 230 bp of sand fly 18S rRNA, respectively. For standardization of our multiplex PCR, different concentrations of SFNGSF and SFNGSR primers (1.0, 0.1, 0.01 and 0.001 µM) were used in the reaction mixture to verify the lowest concentration of primers needed for amplifying the sand fly 18S rDNA fragment. The lowest detection limit of *Leishmania* DNA was determined using tenfold serial dilutions of pure *L. tropica* DNA (1–0.00032 ng), which were added to 10 ng of *Ph. sergenti* DNA and subjected to multiplex PCR. Following optimization, the PCR reaction mixture composed of 12.5 μl of PrimeSTAR Max Premix (2×) (Takara Bio Inc., JP), 1 μM of ITS1NGSF and ITS1NGSR primers, and 0.1 μM of SFNGSF and SFNGSR primers and 2.5 μl of DNA template for a final volume of 25 μl. To validate our assay, 17 DNA reference samples (eight sand fly DNA samples and nine *Leishmania* DNA samples) were tested (Additional file 1: Table S1), and then all field-collected female sand flies were subjected to multiplex PCR for simultaneous detection and identification of sand flies and *Leishmania* parasites.

### DNA library preparation

The multiplex PCR products (25 μl) were cleaned by AMPure XP beads, Beckman Coulter (X1), and eluted in 25 μl elution buffer. The purified products (7.5 μl) were subjected to a second PCR to include unique index sequences (N7XX and S5XX) for barcoding of each sample using Nextera XT Index Kit (Illumina, San Diego, CA, USA). Then, 5 μl from each barcoded sample was pooled together, mixed and spun down. Finally, 100 μl of pooled DNA was purified using X1 AMPure XP beads and quantified by Qubit® Fluorometer (Invitrogen) machine. The concentration of 4.0 nM was prepared from the pooled sample. At least 10,000 reads for each sample were targeted. Samples were deep-sequenced with the Nextseq500 machine using the 150-cycle mid output kit (Illumina, Inc., USA) from the forward read direction.

### Bioinformatics analysis

Binary Base Call (BCL) output files from a Nextseq 500 machine were converted to FASTQ format, using BCL to FASTQ (bcl2fastq v2.20.0.422 Copyright (c) 2007–2017 Illumina, Inc.). The files were analyzed using the Galaxy program (Galaxy Version 0.7.17.1) (https://usegalaxy.eu/). Initially, the obtained sequences were run through fastqc (https://www.bioinformatics.babraham.ac.uk/projects/fastqc/) to check the quality of the generated reads. The sequences were then trimmed (https://usegalaxy.org/) using default parameters to retain the highest quality reads (reads above 100 bp) and a minimum quality score of > 20, which represents an error rate of 1 in 100 (according to Illumina Nextseq machine sequencing error rate), with a corresponding call accuracy of 99%. The filtered data were captured by virtual specific sand fly and *Leishmania* probes at the genus and species levels. Based on the species-specific probes, the number of sequence reads for each species was determined. When non-*Leishmania* or non-sand fly species were detected, the dominant sequence was megablasted (https://blast.ncbi.nlm.nih.gov/Blast.cgi) to confirm the species. The cutoff value of 200 reads per sample was set up to determine *Leishmania* positivity as described previously [45].

### Statistical analysis and phylogenetic tree

Statistical analysis was performed using Statistical Package for the Social Sciences (SPSS) version 20.0 (IBM Corp.) to test the Spearman’s correlation to measure the degree of correlation between classical microscopically results and the Amp-NGS results. GraphPad free online program (https://www.graphpad.com/quickcalcs/kappa2/) was used to calculate the degree of agreement (kappa). The free online program was used for kappa agreement tests. Cohen’s kappa coefficient (k) is a measure of the agreement between two tests beyond that expected by chance, where 0 is chance agreement and 1 is perfect agreement for the measure the degree of association between classical microscopically results and performance against the Amp-NGS results assay. The significance tests were two-tailed. The differences were considered statistically significant when *P*-values were < 0.05.

Phylogenetic tree construction was carried out using the statistical method maximum likelihood (ML) with a bootstrap value of 1000 replications using MEGA X program [46]. Partial DNA sequences of 18S rDNA (150 bp) and ITS1 (150 bp) were used to build the phylogenetic tree based on complete deletion option with gaps and missing data were eliminated. These two 150-bp sequences were produced by the Illumina nextseq500 sequencing machine using the 150-cycle mid output kit (Illumina, Inc., USA). All were based on the Jukes‐Cantor model for nucleotide sequences. Initial tree for the heuristic search was automatically obtained by applying the nearest‐neighbor‐interchange (NNI) algorithms to a matrix of pairwise distances estimated using the maximum composite likelihood (MCL) approach.

## Results

### Morphological identification of sand flies

A total of 187 female sand flies were collected from Tubas, northern Palestine, a focus of CL caused by *L. tropica*, and from Bet Oula, Al-Khalil, southern Palestine (endemic for VL caused by *L. infantum*). All sand flies were identified morphologically as belonging to nine species (Table 2). The most abundant species were *Ph. sergenti* (*n* = 143, 76.5%) and *Ph. papatasi* (*n* = 21, 11.2%), representing together 87.7% of the sand flies collected.

**Table 2:** Microscopic identification of field-collected sand flies from Tubas and Bet Oula, 2018

Sand fly species	Number (%)
*Ph. sergenti*	143 (76.5)
*Ph. papatasi*	21 (11.2)
*Ph. perfiliewi*	6 (3.2)
*Ph. tobbi*	5 (2.7)
*S. dentata*	4 (2.1)
*S. fallax*	2 (1.1)
*S. theodori*	3 (1.6)
*S. tiberiades*	2 (1.1)
*S. christophers*	1 (0.5)
Total	187 (100)

### Sensitivity and specificity of the multiplex PCR assay

**Fig. 3:**
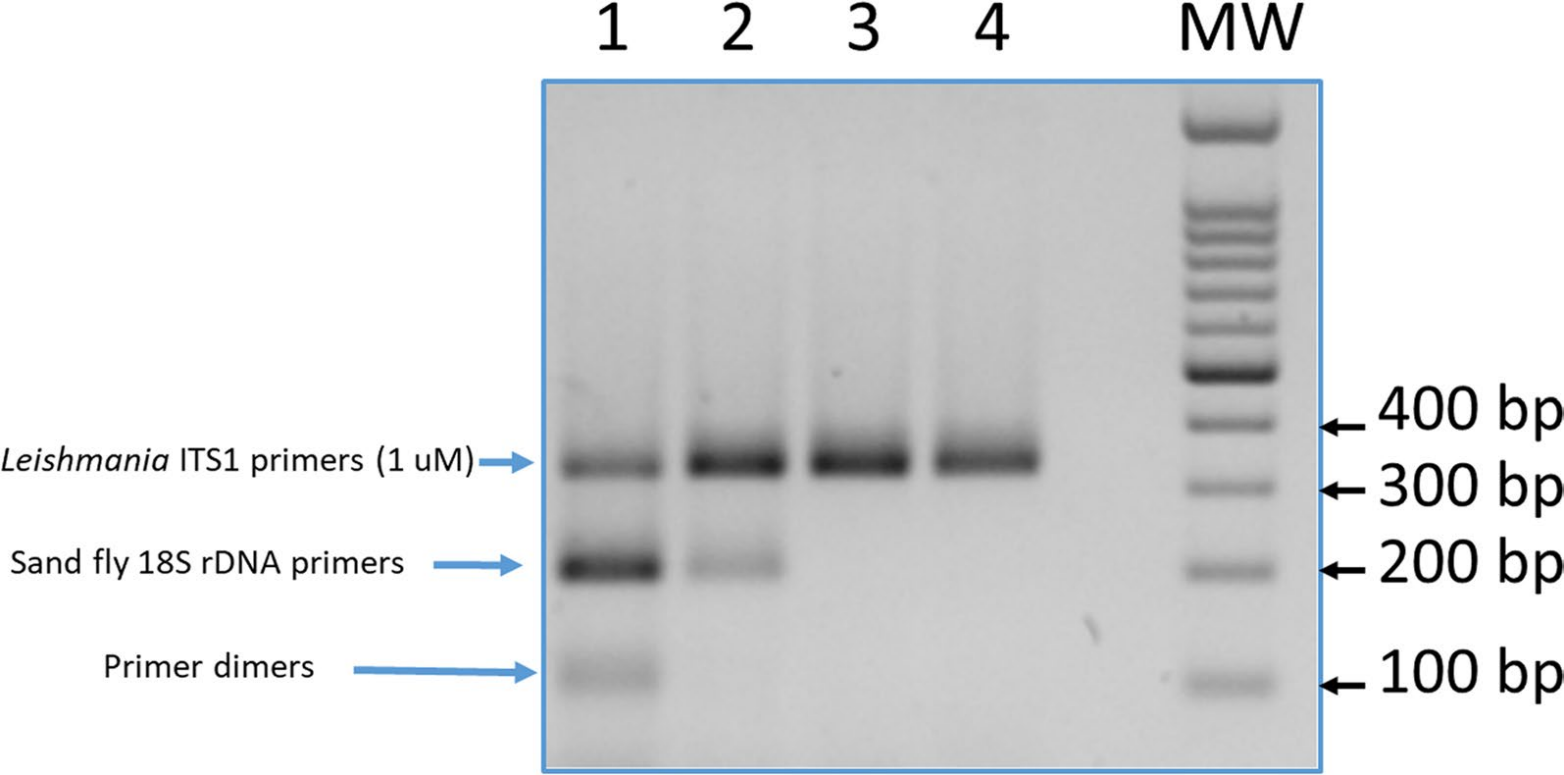
A 1.5% agarose gel obtained from the amplification of partial fragments of sand fly 18srDNA and *Leishmania* ITS1 genes with the multiplex PCR assay using different concentrations of 18SrRDNA sand fly primers (1–4: 1, 0.1, 0.01 and 0.001 µM) and 1 µM of ITS1 primers

Before optimizing the multiplex PCR assay, two singleplex PCRs were performed to detect the sand fly and *Leishmania* DNA utilizing reference DNA samples. The two sets of primers specific for sand flies (SFNGSF/SFNGSR) and *Leishmania* (ITS1NGSF/ITS1NGSR) amplified the expected targets without showing any signs of non-specific bands (data not shown). After testing different concentrations of the sand fly 18S rDNA primers (SFNGSF/SFNGSR) and the *Leishmania* ITS1 primers (ITS1219NGSF/ITS1219NGSR) for the best optimal primer concentration which allowed the amplification of 0.4 ng of *L. tropica*, DNA was 0.1 µM and 1 µM, respectively. (Fig. 3).

The analytical sensitivity of the Amp-NGS assay for *Leishmania* DNA was 0.0016 ng/µl corresponded to 493 reads of ITS1 Leishmanial DNA sequences obtained by the Nextseq500 sequencing machine at a constant concentration of 10 ng of *Ph. sergenti* DNA, which corresponded to 192,389 reads of sand fly DNA sequence of 18S rDNA (Table 3). The optimized multiplex PCR was then applied to 187 field-collected sand flies producing the expected amplicon size of ~ 230 bp. Due to Illumina technology using the 150-cycle the Nextseq500 machine and mid output kit (Illumina, Inc., USA), only 150 bp was sequenced from the forward read direction.

**Table 3:** Serial concentrations of *Leishmania* DNA with their concomitant number of reads for sand flies and *Leishmania* parasites

*Leishmania* DNA concentration (ng)	Sand fly number of reads^a^	*Leishmania* number of reads
1	106,957	19,617
0.2	110,496	17,037
0.04	129,792	5770
0.008	175,327	3262
0.0016	192,389	493
0.00032	206,048	0
0	199,748	0

^a^At DNA concentration of 10 ng

**Fig. 4:**
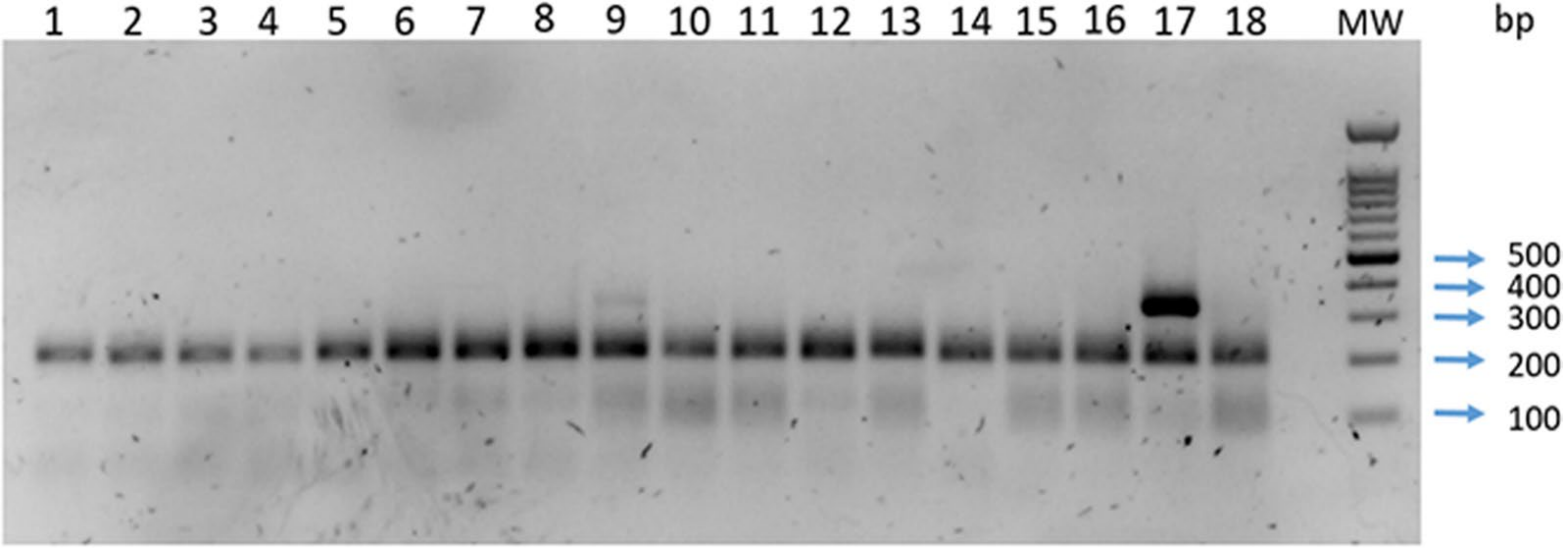
A 1.5% agarose gel obtained from the amplification of 18 field-collected sand flies targeting the sand fly 18S rDNA and *Leishmania* ITS1 with multiplex PCR assay. Concentrations of 0.1 µM of 18S rDNA and 1 µM of ITS1 primers were used. MW is a 100-bp marker

As shown in Fig. 4, the lower line of bands from 1 to 18 at the size of 230 bp represented amplified DNA identical to sand fly DNA, while the upper line of bands at the size of 343 bp represented amplified DNA identical to *Leishmanial* DNA, which is in this case lanes 9 and 17 only.

### Sand flies and *Leishmania* parasites detection by Amp-NGS

To validate Amp-NGS for identification of *Leishmania* and sand fly DNA, DNA from reference samples (sand flies and *Leishmania*) was mixed in a 1:1 volume ratio and subjected to multiplex PCR. A DNA library was prepared, cleaned and sent for deep sequencing as described above. Based on our workflow analysis, the used virtual probes specific for *Leishmania* (Additional file 1: Table S3) correctly identified all *Leishmania* reference DNA samples to the species level. Similarly, reference sand fly DNA samples (Additional file 1: Table S1) were correctly identified to the species level using species-specific virtual probes, which included the species of *Ph. sergenti, Ph. perfiliewi, Ph. syriacus, Ph. papatasi, Ph. tobbi, Ph. argentipes, L. guyanensis* and *S. schwetzi.* To assess the practical usability of Amp-NGS, the assay was applied on field-collected sand flies. Out of 187 tested sand flies, 177 (95%) belonged to *Phlebotomus* spp. including the two major species, *Ph. sergenti* (*n* = 147, 78.6%) and *Ph. papatasi* (*n* = 19, 10.2%), whereas only 5% (10/187) belonged to *Sergentomyia* spp. including *S. dentata* (*n* = 6), *S. fallax* (*n* = 2), *S. ghesquiere* (*n* = 1) and *S. schwetzi* (*n* = 1) (Table 4). The study observed strong positive Spearman’s correlation between sand fly identification results of the Amp-NGS and morphological identification method (*r* = 0.84, df = 185, *P* < 0.001).

**Table 4:** Comparison between the Amp-NGS method and the morphological method for identification of sand fly species

	*Ph. sergenti*	*Ph. papatasi*	*Ph. syriacus*	*Ph. perfiliewi*	*Ph. tobbi*	*S. dentata*	*S. fallax*	*S. ghesquiere*	*S. schwetzi*	Total
*Ph. sergenti*	143	0	0	0	0	0	0	0	0	143
*Ph. papatasi*	2	19	0	0	0	0	0	0	0	21
*Ph. perfiliewi*	0	0	3	3	0	0	0	0	0	6
*Ph. tobbi*	0	0	3	0	2	0	0	0	0	5
*S. dentata*	0	0	0	0	0	4	0	0	0	4
*S. fallax*	0	0	0	0	0	2	0	0	0	2
*S. theodori*	1	0	0	0	0	0	2	0	0	3
*S. tiberiades*	1	0	0	0	0	0	0	1	0	2
*S. christophers*	0	0	0	0	0	0	0	0	1	1
*Total*	147	19	6	3	2	6	2	1	1	187

Concordance between tests was determined using the kappa index (k), which was 0.976 (kappa between 0.81 and 1.00 indicates perfect agreement) with standard error (SE) of 0.028 (95% CI 0.906 to 1.000). The two methods were in perfect agreement in 173 sand flies, but discrepant in 14 sand flies (Table 4). All *Ph. sergenti* (*n* = 143) and 19 *Ph. papatasi* were correctly identified by both methods with complete agreement. However, two *Ph. sergenti* revealed by NGS were identified as *Ph. papatasi* by microscopic examination. Further discrepancies in the identification of *Ph. perfiliewi, Ph. tobbi, Ph. syriacus*, and *S. fallax* were found (Table 4). *Leishmania* DNA was detected and identified as *L. tropica* in 14 samples (14/187, 7.5%). All *L. tropica*-positive sand flies were *Ph. sergenti* collected from Tubas.

To confirm the NGS sequencing, Sanger sequencing was performed for two full ITS1 regions and showed similar results to those obtained by Amp-NGS assay.

### Phylogenetic analysis

**Fig. 5:**
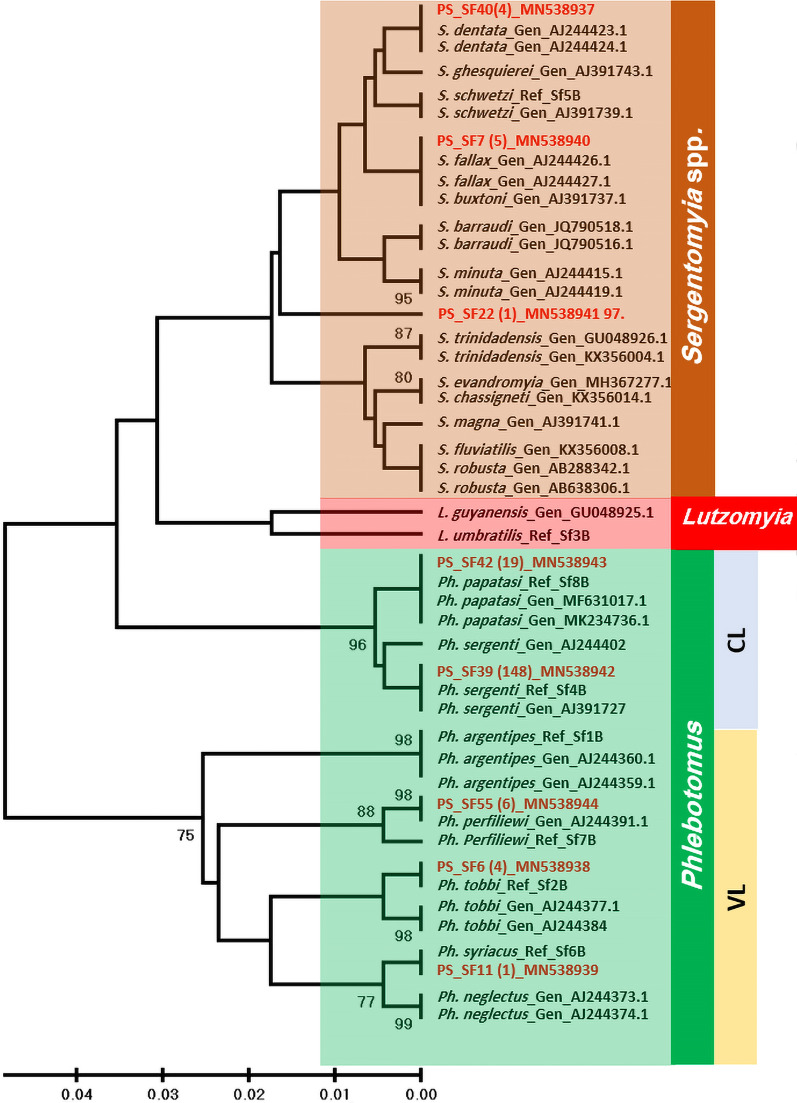
Neighbor-joining (NJ) tree showing the relationships of the study and reference sandflies (*n* = 227) shown in bold red based on 150 bp of the 18srDNA gene sequences. MEGA X program was used for constructing the phylogenetic trees. DNA sequences were aligned using Clustal-W program. Bootstrap values are based on 1000 replicates [46]. Ph., *Phlebotomus*; S. *Sergentomyia*; L., *Lutzomyia*; Gen_AJ244374.1, GenBank accession number; Ref_sf6B, reference strain_sand fly number 6B; CL, cutaneous leishmaniasis; VL, visceral leishmaniasis; PS, Palestine with Palestinian sand fly code; numbers in parentheses indicate number of samples with identical genetic characters

Sand fly 18S rDNA sequences (150 bp) obtained from Amp-NGS assay were used to study the phylogenetic relationships between 187 field-collected sand flies included in this study and 31 sequences retrieved from the GenBank representing different species of sand flies obtained from different geographic regions. Nine DNA sequences representing the sand fly controls used in the study were also included in the phylogenetic analysis. The phylogenetic tree (*n* = 227) demonstrated three clearly separated clusters, *Phlebotomus* and *Sergentomyia*, the Old World sand flies, and *Lutzomyia*, the New World sand flies (Fig. 5). The phylogenetic analysis showed that the geographical origin did not have any effect on the clustering of the 18S rDNA sequences (Fig. 5). It was notable that the nucleotide 18S rDNA sequences of the four *S. dentata* identified in this study were identical to each other and to the respective *S. dentata* reference sequence (accession no. AJ244423.1) obtained from Greece. Furthermore, the 18S rDNA sequences of *Ph. sergenti* (*n* = 147) and *Ph. papatasi* (*n* = 19), CL vectors, formed two related sub-clusters within the *Phlebotomus* clade, while VL vectors, *Ph. neglectus, Ph. perfiliewi, Ph. tobbi* and *Ph. syriacus* were closely related to *Ph. argentipes,* another VL vector (Fig. 5).

## Discussion

Herein, we used a unique amplicon-based multiplex NGS method to concurrently identify species of wild-caught sand flies and detect naturally infecting *Leishmania* species. The uniqueness of this method, to our knowledge, comes from use of a one-step reaction to identify the species across both sand fly and *Leishmania* genera from a single sand fly. To date, most conventional molecular biology approaches were developed to identify *Leishmania* parasites in hosts or vectors by targeting different genes [23, 25–27, 32, 47–52]. The limit of detection (LoD) of these methods ranged from three promastigotes to less than one promastigote in the tested specimen [22, 23, 25–27, 32]. PCR-based methodologies have been developed further into multiplex PCR followed by either restriction fragment length polymorphism(RFLP) or dot blot hybridization for further identification [31, 44, 53–55]. The Amp-NGS in this study was able to detect *Leishmania* spp. and identity sand fly from a single midgut without needing any extra confirmatory or genotyping methods.

Recently, real-time PCR (qPCR) was used to identify *Leishmania* spp. in sand fly employing kDNA, spliced-leader RNA or vacuolar ATPase subunit C (VATP) with a LoD of 1 fg of DNA, 10^–3^ of a parasite and 0.162 parasite, respectively [33, 56–60]. The Amp-NGS was successfully able to detect both *Leishmania* and sand fly at a minimum concentration of 0.0016 ng (< 1 parasite) (Table 3) without any cross-hybridization between the two primer pairs and any mispriming with the other template [61]. Another study used high-resolution melt (HRM) qPCR to detect *Leishmania* [33]. Hitherto, only a handful of studies used NGS technology in the identification of *Leishmania* that used ITS1 and HSP70 (heath shock protein) with the former using longer targets (500 bp) and ignoring validity checks, thus reducing sensitivity and specificity [36, 62, 63].

In this study, multiplex Amp-NGS method was proven to be highly *Leishmania*-specific by correctly identifying the five *Leishmania* spp. controls from France, Ethiopia, Italy, India, Palestine and Iraq. At the same time, the method was highly sand fly-specific by correctly distinguishing the three genera of sand flies, *Phlebotomus*, *Sergentomyia* and *Lutzomyia*, as well as identifying the six *Phlebotomus* species controls.

Amp-NGS for both *Leishmania* and sand fly analyses depends on the concept of sequencing hundreds to thousands of the same amplified DNA molecules (depth) followed by generating a collapsed DNA sequence out of multiple sequences (reads) into one reliable sequence that is compared to a known reference sequence. The bioinformatics analysis of these hundreds to thousands of sequences (reads) of the same target will suppress sequence errors and performs deduplications to produce a very specific and accurate collapsed sequence. In addition, an incorrect base call probability of 1 in 100 produced sequences [lower base call accuracy of 99% (Q20)] with read length < 100 bp were removed from the analysis [64]. Moreover, our study used common and species-specific virtual probes in the polymorphic regions of the targeted sequences that ensured the validity (accuracy) and reliability (repeatability and consistency) of sand fly and *Leishmania* parasite identification.

The sand fly species identified by Amp-NGS were in perfect agreement with the morphological identification as the reference method [kappa index (*k*) = 0.976], indicating high reliability of the Amp-NGS (Table 4). The six discrepant samples were identified as *Ph. syriacus* by Amp-NGS, while by morphological examination, three of them were *Ph. perfeliewi* and the other three were *Ph. tobbi.* Both species belong to the subgenus *Larroussius*, which are morphologically hardly distinguishable (personnel communication). Similarly, some samples of *Sergentomyia fallax*, *S. dentata* and *S. theodori* species were interchangeably identified by the morphological method. The separation of these species is mainly based on differences in shape and armature of the structures of mouthparts and the pharynx. It is reported that some sand flies belonging to the same genus do not comply with all known morphological features, leading to misidentifications [65, 66]. The few discrepant cases between morphological and AMP-NGS could be due to missing a few recently written keys or drawings in morphological identification that could lead to the misidentification of species.

NGS is superior to Sanger sequencing in several aspects. NGS is significantly quicker, lower DNA concentration is required, and it is more accurate and reliable compared to Sanger sequencing. Furthermore, NGS has higher throughput with lower cost per sample. Unlike Sanger sequencing, NGS is able to identify contaminated DNA of more than one target in a single reaction.

Using phylogenetic analysis the Amp-NGS efficiently grouped sand flies into clusters matching the scientifically known sand fly taxonomic units on the levels of genera and species. All GenBank-retrieved and study sand flies clustered into three main genera (*Phlebotomus*, *Sergentomiya* and *Lutzomyia*). The genus *Phlebotomus* was further divided into two sub-clusters, CL and VL (Fig. 5). The congruence between the sand fly species detection by Amp-NGS and morphological identification on one side and the agreement between Amp-NGS and phylogenetic analysis on the other confirms the reliability of Amp-NGS as an identification method.

Here, despite a relatively small size of samples tested (*n* = 187), the main dominant genus of the sand fly in Palestine was *Phlebotomus* spp. (95%) compared to the low prevalent *Sergentomyia* (5%). *Phlebotomus* spp. are the main incriminated vectors of *Leishmania* parasite [41]. Out of the 95% *Phlebotomus* spp., 83% were *Ph. sergenti*, the *L. tropica* vector, and 11% were *Ph. papatasi*, the *L. major* vector, which concords with a previous study [8].

## Conclusions

Our newly developed Amp-NGS assay was optimized and validated as a powerful tool for the simultaneous identification of sand flies and *Leishmania* species. This assay allows the identification of large numbers of sand flies, which may be useful for large-scale field studies.

## Supplementary Information

**Additional file 1: Table S1**. Reference DNA samples for *Leishmania* and sand fly species used in the study.** Table S2.** Sand flies and* Leishmania* GenBank accession numbers used in this study for primer designing. **Table S3.** The virtual probe sequences used for detection and identification of sand flies and *Leishmania* parasites.

## Data Availability

Representative sand fly DNA sequences were deposited in the GenBank under the accession numbers MN538937-MN538944. Two *L. tropica* sequences of about 270 bp detected in two sand flies were also deposited in the GenBank (accession numbers: MT966013-MT966014).

## Abbreviations

CL: Cutaneous leishmaniasis
VL: Visceral leishmaniasis
Amp-NGS: Amplicon-based next-generation sequencing
MCL: Mucocutaneous leishmaniasis
hVL: Human visceral leishmaniasis
canL: Canine leishmaniasis
ITS1: Internal transcribed spacer 1
hsp70: Heat shock protein 70
LoD: Limit of detection
HRM: High-resolution melt
RFLP: Restriction fragment length polymorphism
spp: Species
