# Supplementary material for: Concurrent molecular characterization of sand flies and Leishmania parasites by amplicon-based next-generation sequencing
Source: Parasit Vectors. 2022 Jul 22;15:262. doi: 10.1186/s13071-022-05388-3 (PMC9308317; doi:10.1186/s13071-022-05388-3)
Supplement: Supplementary file 1 — Additional file 1: Table S1. Reference DNA samples for Leishmania and sand fly species used in the study. Table S2. Sand flies and Leishmania GenBank accession numbers used in this study for primer designing. Table S3. The virtual probe sequences used for detection and identification of sand flies and Leishmania parasites. [file 13071_2022_5388_MOESM1_ESM.docx]

**Table S1**: Reference DNA samples for *Leishmania* and sand fly species used in the study.

| ***Leishmania* WHO code** | ***Leishmania* spp.** | **Sand fly species** |
| --- | --- | --- |
| MPRO/IL/2003/HYRAX107 | *L. tropica* | *Ph. sergenti* |
| MHOM/IL/2???/LRC-L1686 | *L. tropica* | *Ph. perfiliewi* |
| MHOM/IL/2005/LRC-L1188 | *L. tropica* | *Ph. syriacus* |
| MHOM/ET/1972/L102 | *L. aethiopica* | *Ph. papatasi* |
| MHOM/SU/1973/5ASKH | *L. major* | *Ph. tobbi* |
| MHOM/IL/2003/LRC-L949 | *L. major* | *Ph. argentipes* |
| MHOM/IL/1998/LRC-L745 | *L. infantum* | *Lutzomyia spp.* |
| MCAN/IT/2007/LRC-L1314 | *L. infantum* | *Sergentomyia* genus |
| MHOM/ET/????/HU3 | *L. donovani* |  |

**Table S2**. Sand flies and *Leishmania* GenBank accession numbers used in this study for primer designing.

| **Sand flies** |  | ***Leishmania*** |  |
| --- | --- | --- | --- |
| **GenBank Accession Number** | **Species** | **GenBank Accession Number** | **Species-country code** |
| AJ391727 | *Ph. sergenti* 1 | AJ276259.1 | Ldon_SD |
| GU048925.1 | *L. guyanensis* | AJ000292.1 | Ldon_IN |
| AJ391743.1 | *S. ghesquiere* | AM901452.1 | Ldon_IQ |
| AJ244424.1 | *S. dentata* | MH450081.1 | Ldon_IN |
| AJ244423.1 | *S. dentata* | AJ000289.1 | Linf_TN |
| JQ790516.1 | *S. barraudi* | MK675913.1 | Linf_FR |
| AJ391737.1 | *S. buxtoni* | KU680854.1 | Ltrop_IR |
| AJ244427.1 | *S. fallax* 1 | EU683617.1 | Ltro_IL |
| AJ244426.1 | *S. fallax* 2 | FN677341.1 | Ltro_IL |
| AJ244419.1 | *S. minuta* 1 | FN677354.1 | Laeth_ET |
| AJ244415.1 | *S. minuta* 2 | FN677346.1 | Laeth_ET |
| KX356008.1 | *S. fluviatilis* | KF981809.1 | Lmaj_CM |
| AB638306.1 | *S. robusta* 1 | KY882278.1 | Lmaj_IQ |
| AB288342.1 | *S. robusta* 2 |  |  |
| MH367277.1 | *S. evandromyia* |  |  |
| KX356014.1 | *S. chassigneti* |  |  |
| KX356004.1 | *S. trinidadensis* 1 |  |  |
| GU048926.1 | *S. trinidadensis* 2 |  |  |
| AJ391741.1 | *S. magna* |  |  |
| MF631017 | *Ph. papatasi* 1 |  |  |
| MK234736.1 | *Ph. papatasi* 2 |  |  |
| AJ244377.1 | *Ph. tobbi1* |  |  |
| AJ244360.1 | *Ph. argentipes* 1 |  |  |
| AJ244359.1 | *Ph. argentipes* 2 |  |  |
| AJ244384 | *Ph. tobbi* 2 |  |  |
| AJ244374.1 | *Ph. neglectus* 1 |  |  |
| AJ244373.1 | *Ph. neglectus* 2 |  |  |
| AJ244389 | *Ph. perfiliewi* 1 |  |  |
| AJ244391.1 | *Ph. perfiliewi* 2 |  |  |

**Table S3.** The virtual probe sequences used for the detection and identification of sand flies and *Leishmania* parasites.

|  | | **SF species** | **Specific probe (5’ to 3’)** | |
| --- | --- | --- | --- | --- |
| 1 | | Sand fly (common) | TGCGGTTAAAACGTTCGTAG | |
| 2 | | *S. dentata* | ACACGGGCAATGCAC | |
| 3 | | *S. barraudi* | TTTGTGCATTGGTAAAACAGTGTGCAA | |
| 4 | | *S. minuta* | AATGACTTTA | |
| 5 | | *Ph. Sergenti* | GCTCTGTGCGTTTTGTGTA | |
| 6 | | *Ph. perfiliewi* | CGCATATGTTTCACCGTA | |
| 7 | | *Ph. perfiliewi* | TACTATATGTTCACCGTCA | |
| 8 | | *Ph. neglectus* | TCGCATATGTGTGTCTCACCGTCA | |
| 9 | | *Ph. sergentomyia* | CAGTGTGCAG | |
| 10 | | *Ph. papatasi* | CTGTGCGTTCTGTGTAAAAGCAAGCGTATAGT | |
| 11 | | *Ph. syriacus* | TCGCATATGTGTGTCTCACCGTAA | |
| 12 | | *Lutzomyia* | ACTTGTTGCCT | |
| 13 | | *Lutzomyia umbratilis* | AAACAGTGTGTAT | |
| 14 | | *Ph. argentipes* | TCAAAGGTGG | |
| 15 | | *Ph. tobbi* | AAAGTGTCATATGTATGT | |
|  |  | | |  |
|  | ***Leishmania* species** | | | **Specific probe** |
| 1 | *Leishmania* (common) | | | GATCATTTTCCGATG |
| 2 | *L. major* | | | TTTTATACTCAAAATTTGCA |
| 3 | *L. donovani* | | | ATTACACCAAAAAA |
| 4 | *L. tropica* | | | CATATACAAAACTCGGGGAGGCCTAT |
| 5 | *L. infantum* | | | CGTTATAACGCA |
| 6 | *L. atheiopica* | | | TCGGGCAGGCCTATTA |
| 7 | *L. tropica* | | | ATTACACCCCAAAAAAAACA |
